# Diagnostic Utility of Clinical Neurophysiology in Jerky Movement Disorders: A Review from the MDS Clinical Neurophysiology Study Group

**DOI:** 10.1002/mdc3.14306

**Published:** 2024-12-18

**Authors:** Anna Latorre, Christos Ganos, Masashi Hamada, Nicolas Phielipp, Lorenzo Rocchi, Shabbir Merchant, Marina A. Tijssen, Sterre van der Veen, Robert Chen

**Affiliations:** ^1^ Department of Clinical and Movement Neurosciences, UCL Queen Square Institute of Neurology University College London London United Kingdom; ^2^ Movement Disorder Clinic, Edmond J. Safra Program in Parkinson's Disease, Division of Neurology University of Toronto, Toronto Western Hospital Toronto Ontario Canada; ^3^ Department of Neurology The University of Tokyo Tokyo Japan; ^4^ Department of Neurology, Parkinson's and Movement Disorders Program University of California Irvine Irvine California USA; ^5^ Department of Medical Sciences and Public Health University of Cagliari Cagliari Italy; ^6^ Department of Neurology, Beth Israel Deaconess Medical Centre, Harvard Medical School Boston Massachusetts USA; ^7^ Department of Neurology, University of Groningen, University Medical Centre Groningen (UMCG) Groningen The Netherlands; ^8^ Expertise Centre Movement Disorders Groningen University Medical Centre Groningen (UMCG) Groningen The Netherlands; ^9^ Krembil Research Institute, University Health Network University of Toronto Toronto Ontario Canada; ^10^ Division of Neurology, Department of Medicine University of Toronto Toronto Ontario Canada

**Keywords:** myoclonus, myoclonus dystonia, brainstem myoclonus, startle, clinical neurophysiology, jerky movement disorders

## Abstract

**Background:**

Myoclonus and other jerky movement disorders are hyperkinetic disorders, the diagnosis of which heavily relies on clinical neurophysiological testing. However, formal diagnostic criteria are lacking, and recently the utility and reliability of these tests have been questioned.

**Objective:**

The aim of this review was to assess the utilization of clinical neurophysiology testing to identify possible gaps and boundaries that might guide the development of new methods for a more precise diagnosis and in‐depth understanding of myoclonus.

**Methods:**

We reviewed electrophysiological features of cortical myoclonus, subcortical myoclonus (ie, myoclonus associated with dystonia, brainstem myoclonus), excessive startle reflex, spinal myoclonus (ie, spinal segmental and propriospinal myoclonus), peripheral myoclonus and mimics of myoclonus of peripheral origin (hemifacial spasm, minipolymyoclonus, myokymia), functional jerky movements, chorea, and tics.

**Results:**

Electrophysiological features that support the recognition of myoclonus subtypes, such as muscle burst duration, muscle pattern of activation, measures of cortical excitability, or movement‐related cortical potentials, have been identified. These significantly contribute to the diagnosis of jerky movement disorders, but their reliability is uncertain. Despite the significant advancements, several unresolved questions persist. Factors contributing to this include the absence of systematic neurophysiological assessment and standardized methods, alongside the limited number of patients investigated using these techniques.

**Conclusion:**

Although clinical neurophysiology remains the “gold standard” for defining and diagnosing myoclonus, our review highlighted the need to enhance the quality and reliability of neurophysiological testing in jerky movement disorders. Further studies including larger cohorts of patients recruited from different centers, employing standardized and optimized electrophysiological techniques, are warranted.

Myoclonus and other jerky movement disorders are hyperkinetic disorders, the diagnosis of which heavily relies on clinical neurophysiology (CN) testing.^1^, ^2^, ^3^ This includes techniques such as surface poly‐electromyographic (EMG) recording of myoclonic jerks, long‐latency EMG responses to mixed or cutaneous nerve stimulation (long‐latency reflex [LLR]), electroencephalography (EEG), EEG–EMG polygraphy using back‐averaging (including Bereitschaftspotential [BP]), and cortico‐muscular coherence analysis, as well as somatosensory evoked potentials (SEP).^4^, ^5^ These tests not only aid the differential diagnosis between myoclonus and other jerky movement disorders, and in certain cases help identify their underlying causes (eg, BP for functional jerky movement disorders), but can also identify the anatomical source of the myoclonus within the nervous system. According to this principle, four main myoclonus subtypes can be distinguished: cortical, subcortical (including myoclonus associated with dystonia, brainstem myoclonus), spinal (including segmental and propriospinal), and peripheral. An alternative classification of myoclonus has been proposed,^6^ which, apart from cortical and peripheral, comprises the categories of cortical–subcortical, subcortical nonsegmental myoclonus, and segmental.^7^ In this review we used the first classification as it is more applicable to clinical practice and more accurately reflects the pathophysiology of myoclonus and the neurophysiological findings associated with this disorder.

Beyond the classification, there is also a need to establish definitive CN diagnostic criteria for myoclonus. Despite being deemed the “gold standard,” mainly to differentiate cortical from non‐cortical myoclonus,^2^, ^5^, ^8^ the diagnostic utility of CN has been called into question, due to several limitations.^9^, ^10^ For instance, retrospective studies reported that CN testing revealed a cortical origin only in a minority of patients presumed to have cortical myoclonus clinically,^9^, ^11^ whereas the diagnostic accuracy of CN remains inadequately investigated.^10^ Moreover, there is a lack of consensus on CN criteria, which are inconsistently applied across studies.

To elucidate the diagnostic value of CN in myoclonus, we conducted a thorough review of the electrophysiological features utilized to diagnose and differentiate various myoclonus subtypes, including mapping key milestones in the application of neurophysiological techniques to this disorder. Furthermore, we reviewed the application of CN for the differential diagnosis of myoclonus versus other jerky movement disorders, including excessive startle, mimics of myoclonus of peripheral origin, functional jerky movements, chorea, and tics, which often pose diagnostic challenges for clinicians.

Our aim was to identify possible gaps and boundaries that might guide the development of new methods for a more precise diagnosis and in‐depth understanding of these disorders.

## Patients and Methods

We identified 6 topics for literature search: (1) cortical myoclonus, (2) subcortical myoclonus (including myoclonus associated with dystonia, brainstem myoclonus) and excessive startle reflex, (3) spinal myoclonus (including spinal segmental and propriospinal myoclonus), (4) peripheral myoclonus and mimics of myoclonus of peripheral origin (such as minipolymyoclonus, myokymia, hemifacial spasm), (5) functional jerky movements, and (6) tics and chorea. Each of these topics was assigned to 2 members of the *myoclonus and other jerky movements group* of the Movement Disorders Society CN Study Group for conducting literature review. The findings were presented to the entire group for discussion and analysis. Considering the broad topic, we opted for a narrative, because the definition of a strict search strategy would have limited our findings. Nevertheless, the key words primarily used to select studies on Medline database were cortical, subcortical, brainstem, spinal, propriospinal, peripheral myoclonus, myoclonus dystonia, excessive startle reflex, tics, chorea, functional jerky movements together with neurophysiology, EMG, EEG, back‐averaging, SEP, giant SEP, cortico‐muscular coherence, and BP. Review articles were checked to include relevant articles and information. All types of original articles were included with the following exclusion criteria: written language other than English, use of noninvasive brain stimulation techniques, or other neurophysiological techniques not for diagnostic purpose.

## Results

### Cortical Myoclonus

Professor Friedreich in 1881^12^ first published a case of a man affected by pneumonia, who exhibited brief, arrhythmic, stimulus‐sensitive, multifocal twitches that he called paramyoclonus multiplex. Grinker, Serota, and Stein^13^ observed that EEG abnormalities were related to specific jerky movements in epileptic patients, and Dawson^14^ reported the first recordings of enlarged SEP in patients with myoclonic epilepsy, evoked by tapping tendons or electrical stimulation. Halliday^15^ further characterized the cortical involvement in what he called “pyramidal” myoclonus. In 1975, Shibasaki and Kuroiwa^16^ introduced the EEG back‐averaging technique using the brief muscle twitch as a trigger event, showing that a cortical transient preceding the muscle burst even with an initial unremarkable EEG. Hallett and colleagues^17^ described enhanced cortical(C‐) reflex in patients affected by cortical myoclonus. Reviewing this literature and adding their own observations, Obeso and colleagues^18^ outlined the clinical and neurophysiology spectrum of cortical myoclonus, with neurophysiology comprehensively reviewed by Shibasaki.^19^ Brown and colleagues^20^, ^21^ described abnormal cortico‐muscular and intermuscular coupling in patients affected by high‐frequency rhythmic myoclonus. The main components of cortical myoclonus have been reported from retrospective^9^, ^22^, ^23^ or cross‐sectional^11^, ^20^, ^21^, ^24^, ^25^, ^26^, ^27^, ^28^, ^29^ case series, and a systematic review,^10^ which includes some of the case series referenced before. This literature includes 227 cases in which the clinical features of cortical myoclonus and its physiology correlates have been described (Table 1). Features of negative cortical myoclonus, which refers to a brief and sudden occurrence of EMG silence when holding a posture, have been illustrated^30^ and suggest a cortical origin,^31^ as it can be induced by median nerve stimulation, the same stimulation that can elicit enhanced SEP and C‐reflex in cortical myoclonus. However, most of the published results relate to positive cortical myoclonus (ie, presence of brief muscle contractions). Clinical features of cortical myoclonus include brief movements, focal or more frequently multifocal anatomical distribution, arrhythmic and rarely rhythmic, spontaneous or induced by sensory stimulus or movement, sporadic or familial, primary or secondary. In parallel, the following neurophysiological features have been described for cortical myoclonus: short EMG burst duration, presence of enlarged or giant SEP, enhanced C‐reflex, presence of EEG discharges time locked to individual myoclonic jerks detected with jerk‐locked back‐averaging (JLBA), and enhanced cortico‐muscular and intermuscular coherence. Regarding EMG burst duration, most case series reported durations of ≤50 ms. However, some groups reported cortical myoclonus exceeding this duration up to 200 ms.^18^, ^22^, ^23^ SEPs were studied in 144 of the patients reported (63% of the whole sample), and they were enlarged in 67 subjects (46%). The threshold amplitude to consider SEP to be enhanced was in some studies >12 μV for median nerve stimulation at the wrist, 5.5 μV for finger stimulation, and >2 μV for toe stimulation.^18^, ^32^, ^33^ However, this is laboratory dependent. Case series reviewed usually report higher amplitudes in the patients studied, namely >10 μV measured between P1 (P25) and N2 (N35) in the somatosensory areas. C‐reflex or LLR was studied in 68 subjects (29%), and exaggerated responses were present in 49 subjects (72% of the sample studied). JLBA^16^ was studied in 138 subjects (60%) and was present in 70 subjects (50% of the sample studied). Finally, coherence studies (cortico‐muscular and intermuscular coherence)^11^, ^21^ have been reported to be helpful in patients with rhythmic cortical myoclonus (cortical tremor) with frequency >3 Hz. Cortico‐muscular coherence was studied in 49 patients of the discussed sample and was present in 19 of the patients studied (38%). It is sometimes considered helpful to demonstrate cortical involvement when JLBA does not show positive findings or cannot be applied because of the high frequency of muscle jerks.^21^

**TABLE 1:** Clinical neurophysiological studies in cortical myoclonus

Technique	Subjects studied over a total sample of 227 (%)	Subjects studied showing positive findings (n) and percentage (%)
Jerk locked back‐averaging	138 (60%)	70 (50%)
SEP	144 (63%)	67 (46%)
C‐reflex	68 (29%)	49 (72%)
Cortico‐muscular coherence^a^	49 (21%)	19 (38%)

Abbreviation: SEP, somatosensory evoked potentials.

^a^ Note that this technique can be used only when studying events with a frequency of >3 Hz.

In summary, myoclonus characterized by multifocal, arrhythmic, stimulus‐sensitive, and brief movements with EMG burst durations of <50 ms is highly probable to be of cortical origin. However, longer EMG bursts or rhythmic/focal phenomenology does not rule out cortical myoclonus and necessitates additional assessments such as SEP, C‐reflex, and JLBA. Coherence studies for rhythmic myoclonus exceeding a frequency of 3 Hz are recommended. Regarding the averaging techniques, there is no consistent recommendation for the number of events that need to be averaged, with multiple series reporting >100 events for C‐reflex or JLBA, but ranging between 17 and 200 or more. Despite positive findings in approximately 48% to 72% of cases, the sensitivity of SEP, C‐reflex, and JLBA remains relatively low (Table 1). Uncertainty persists regarding the inconsistent presence of neurophysiological markers across patients with similar clinical presentations, warranting further investigation into the underlying pathophysiology of different cortical myoclonus phenomena and the applied methodologies to capture them. Additionally, discrepancies between clinical and neurophysiological assessments may exist, potentially leading to misclassification. Although individual test sensitivity may be limited, combining tests may improve overall sensitivity.^34^ Finally, further exploration of negative myoclonus is necessary to elucidate the role of the motor cortex in its generation.

### Subcortical Myoclonus

The term “subcortical myoclonus” is employed to differentiate it from cortical myoclonus, which exhibits neurophysiological markers indicative of a cortical origin. Subcortical myoclonus refers to forms of myoclonus where the presumed generator lies between the basal ganglia and the medulla. Following this definition, two primary subtypes can be identified: myoclonus associated with dystonia and brainstem (reticular) myoclonus. Exaggerated startle reflex will also be discussed. Due to the ambiguity surrounding the terms “palatal tremor” and “palatal myoclonus” in the literature, and its current classification as palatal tremor given the rhythmicity of palate movements,^35^ this topic has not been included in this review.

#### Myoclonus Associated with Dystonia

According to several reviews, subcortical myoclonus related to myoclonus dystonia has prolonged burst duration, no stimulus sensitivity, and lack of electrophysiological cortical features compared to cortical myoclonus. Nevertheless, only a few studies have performed myoclonus recording in these patients.^1^, ^4^, ^7^, ^36^ The first study investigating the electrophysiological features of myoclonus dystonia was performed before the discovery of genes associated with this condition.^37^ In 10 patients, EMG, SEP, and EEG were recorded. The findings showed EMG burst durations between 50 and 250 ms, but up to 500 ms, and when the EMG bursts were prolonged, they always produced visible muscle jerks that were distinct from dystonic spasms. The jerks involved one muscle and its synergists or showed co‐contraction of agonist–antagonist muscle groups; they could be rhythmic at time with a frequency of 3 to 4 Hz. Negative myoclonus was observed in some cases. Its duration normally varies from 50 to 500 ms,^30^, ^38^ but in a recent study on progressive myoclonus ataxia, it was found to be from 88 to 194 ms in 6 patients, including 2 with cortical negative myoclonus.^39^ SEP was normal in all myoclonus‐dystonia subjects, except 1 case that showed an amplitude of ~13 μV for the P26‐N35 component contralateral to the affected arm. No EEG activity time locked to the jerks was found in the 8 patients studied using back‐averaging.

Other studies on different myoclonus dystonia syndromes showed similar findings (45 patients in total, but not all underwent all the tests).^40^, ^41^, ^42^, ^43^, ^44^ EMG burst durations were between 15 and 250 ms (1 study reported up to 750‐ms burst duration, likely related to dystonia^40^). The EMG bursts were mostly irregular but sometimes rhythmic^42^ or associated with tremor.^41^ SEP was not enlarged, JLBA was negative (subcutaneous single‐use needle recording in 1 study^42^ and average of 24 epochs in another study^44^), and LLR (recorded under muscle activation^40^, ^42^ and/or at rest) was normal or not found. Only one study investigated EMG–EEG and EMG–EMG coherence in 20 patients (5 of whom were asymptomatic) with myoclonus dystonia caused by *SGCE* gene mutation.^45^ High EMG–EEG coherence was found only in patients with predominant dystonia, but not in patients in whom the predominant feature was myoclonus, whereas there was no EMG–EMG coherence (between the neck muscles, between several arm muscles, and between the arm and neck muscles) at rest and during weak (25% maximum voluntary contraction) isometric contraction when rotating the head or extending the wrist.

In summary, about 50 patients with myoclonus dystonia have been investigated using clinical electrophysiological techniques (Table 2). EMG recording of the myoclonus showed variable burst duration, which could be <50 to 100 ms and up to 500 ms (which might reflect dystonic spasms). SEP, JLBA, and LLR (exact number of patients not available) were negative, apart from 1 patient who showed enlarged SEP. In all patients EMG–EMG and EEG–EMG analyses did not show coherence related to myoclonus.

**TABLE 2:** Clinical neurophysiological findings of myoclonus associated with dystonia

EMG burst duration (range)	Enlarged SEP	JLBA, LLR, EMG–EMG/EMG–EEG coherence
15–750 ms	2 subjects	None

*Notes*: Total subjects studied are 50; exact number for each test is not available.

Abbreviations: EMG, electromyography; SEP, somatosensory evoked potential; JLBA, jerk‐locked back‐averaging; LLR, long‐latency reflex; EEG, electroencephalography.

#### Brainstem (Reticular) Myoclonus

Brainstem or reticular myoclonus is thought to originate from the lower brainstem or reticular formation, in view of its typical muscle pattern of activation. It was first described by Hallett and colleagues in 1977,^46^ who presented a single case of post‐hypoxic “reticular reflex myoclonus” showing, on clinical electrophysiological studies, a short duration of the EMG bursts (~10–30 ms), with earlier activation of trapezius and sternocleidomastoid (SCM) followed by a rostral and caudal progression; activation of the arms tended to precede the legs, and the cranial nerve musculatures (at least those supplied by the lower cranial nerves) seemed to be activated in ascending order, with a latency that was compatible with the distance of the muscles from the neuraxis. SEP was normal and JLBA was negative. The other recordings were performed on single cases (7 in total) with different etiologies^47^, ^48^, ^49^, ^50^, ^51^, ^52^ but showed similar EMG pattern, namely short EMG burst duration (from 10 to 100 ms, except 1 case up to 150 ms^49^) and early activation of muscles innervated by the accessory nerve followed by the orbicularis oculi and then upper‐ and lower‐limb muscles (with a proximal–distal gradient). In 2 cases, C‐reflex was found.^48^ Interestingly, the conduction velocity through the spinal cord was rapid, differentiating brainstem myoclonus from excessive startle reflex.^46^, ^52^

In conclusion, brainstem myoclonus was recorded only in a small number of patients (Table 3). The EMG recording showed short burst durations and a typical pattern of muscle activation. The C‐reflex was found in 2 cases. The conduction velocity through the spinal pathway was measured in 2 patients (80 and 40 m/s, respectively),^46^, ^52^ but in 1 study the methods were not detailed.^52^

**TABLE 3:** Clinical neurophysiological findings of brainstem myoclonus

EMG burst duration	Pattern of muscle activation	C‐reflex	Rapid conduction velocity
10–100 ms (150 ms in 1 case)	Early‐onset activation of the SCM and trapezius muscles followed by rostral and caudal activation, with onset latencies compatible with the distance from the lower brainstem	Found in 2 subjects	Measured in 2 subjects: 80 and 40 m/s

*Note*: Total subjects studied are 8.

Abbreviations: EMG, electromyography; SCM, sternocleidomastoid muscle.

#### Excessive Startle

Startle is a physiological reflex to unexpected auditory and somesthetic stimulation. It is deemed pathological, known as excessive startle, when certain criteria are met: shorter onset latencies of EMG responses, longer burst durations, persistent responses with repeated stimuli but not strictly reduced habituation, and more consistent activation of limbs and lumbar spinal muscles. Importantly, the pattern of muscle activation in excessive startle is similar to the physiological startle response.^53^, ^54^ Excessive startle reflex has been investigated in several studies for 58 patients^53^, ^54^, ^55^, ^56^, ^57^, ^58^, ^59^ but using different methods. Whereas auditory stimulation was used in all studies, other forms of stimulation to elicit the reflex were used only in some, such as peripheral electrical stimulation of sensory and mixed nerves^53^, ^54^, ^59^ and taps to the body with a tendon hammer.^53^, ^54^, ^57^ Regarding auditory stimulation, different protocols have been applied with various sound intensities and stimulation frequencies (ie, randomly about once every 20 min,^54^ every 45 to 60 s for 20 trials,^53^ every 10 or 60s,^55^, ^56^ either unexpected or at 10‐s intervals,^58^ at random intervals of 2 to 5–10 min^57^, ^59^). Despite the diverse protocols, the evoked responses were consistent, with an early auditory blink reflex response followed by activation of the SCM muscle, and then rostro and caudal progression based on the distance of segmental innervations from the caudal brainstem.^60^ The conduction velocity in spinal efferent pathways is considered moderately slow.^53^, ^54^ In cases with excessive startle response, when compared to the physiological startle reflex, no habituation to repeated stimuli was found.^53^, ^54^, ^58^ However, according to 2 studies, habituation depends on severity of the startle response and may be less prominent than in control cases.^55^, ^56^ Two patients with excessive startle reflex were found to have enlarged or giant SEP.^54^

In conclusion, excessive startle reflex is characterized by a reproducible response, elicited by auditory stimulation or other sensory stimuli. Different from brainstem myoclonus, the early response is an auditory blink reflex, and there is a relatively slow recruitment of caudal muscles, indicating moderate conduction velocity in spinal efferent pathways, and longer EMG burst duration.^61^ Additionally, startle reflex is stimulus induced, whereas brainstem myoclonus may occur spontaneously. However, studies have been conducted on a limited number of patients with varying methodologies. Not all studies confirm reduced habituation of excessive startle reflex. Furthermore, enlarged/giant SEP has been found in 2 patients.^54^

### Spinal Myoclonus

#### Propriospinal Myoclonus

Propriospinal myoclonus is a rare form of myoclonus, characterized by jerks of spinal origin.^62^, ^63^ The first description of propriospinal myoclonus in 3 cases was given by Brown and colleagues.^62^ Flexor arrhythmic jerks of the axial muscles were the typical presentation, often accompanied by jerks of proximal limb muscles. Typically, they are stimulus sensitive and increase in the supine position. The EMG discharge lasted from 50 to 300 ms, rarely 1000 ms or longer. The characteristic pattern of muscle activation can be detected by measuring latencies of recruited muscles, showing that the discharge slowly spread (5–15 m/s) up and down the origin in the spinal cord to involve the rostral and caudal segments, presumably via propriospinal pathway. Neither corresponding EEG discharge nor giant SEP was noted.

Although there are some reports in which spinal cord lesions were present in the case of propriospinal myoclonus,^64^, ^65^, ^66^, ^67^, ^68^, ^69^ suggesting that they are symptomatic (ie, secondary), most patients who presented with propriospinal myoclonus are considered to have a functional movement disorder mainly because of the absence of spinal cord lesions and the presence of BP in many cases.^63^, ^70^, ^71^, ^72^ However, it is still controversial whether idiopathic propriospinal myoclonus exists.^73^ We conclude that propriospinal myoclonus is rare,^22^, ^63^, ^70^ and many cases are likely caused by a functional movement disorder.

#### Segmental Myoclonus

This type of myoclonus is considered extremely rare. Segmental myoclonus is the result of abnormal spontaneous discharges of motor neurons in a limited area of the spinal cord, inducing involuntary rhythmic or semirhythmic (usually 1–3 Hz, ranging 2–600 contractions per min^74^) jerks in a muscle or group of muscles innervated by the affected spinal segment. The durations of EMG discharge varied but were usually between 100 and 500 ms.^75^, ^76^, ^77^, ^78^ Segmental myoclonus is generally not stimulus sensitive, but some cases with stimulus sensitivity have been reported.^77^, ^79^ EEG and SEP were normal. Various possible causes have been reported, including autoimmune,^80^, ^81^, ^82^ Chiari malformation,^83^ cervical spondylosis/myelopathy,^74^, ^84^, ^85^, ^86^ cervical transforaminal epidural steroid injection,^87^ demyelination,^88^ drug,^89^ infection,^90^, ^91^ paraneoplastic,^92^ postinfectious,^93^ tumor,^94^, ^95^ spinal surgery,^96^ and vascular diseases.^97^, ^98^

### Peripheral Myoclonus

The term “peripheral myoclonus” indicates rhythmic or semirhythmic jerk secondary to plexus, nerve, root lesion, or, rarely, anterior horn cell disease. Compared to myoclonus from other generators, it presents with specific electrophysiological features, such as long EMG bursts (100–400 ms) and a pseudorhythmic/rhythmic pattern (1–3 Hz).^99^, ^100^, ^101^ It is interesting to note that long EMG bursts and rhythmicity are commonly found in spinal segmental myoclonus as well.^102^ Consistent with this finding, it has been hypothesized that peripheral and spinal segmental myoclonus may share a common pathophysiology, represented by disinhibition of spinal motor circuits, caused by deafferentation in peripheral myoclonus and direct spinal cord damage in spinal myoclonus.^99^, ^100^, ^101^

These physiological features make this form of peripheral myoclonus substantially different from hemifacial spasm that is considered the most common form of peripheral myoclonus. It is typically caused by arterial compression of the facial nerve at its exit site from the pons but has also been reported in multiple sclerosis with or without a unilateral pontine demyelinating lesion.^103^, ^104^ In hemifacial spasm motor units fire in bursts of up to 40 at frequencies of 200 to 300 Hz. There may be synchronicity between different facial muscles that is consistent with ephaptic transmission between neighboring motor axons.^105^

In summary, although peripheral myoclonus might be difficult to distinguish from spinal segmental myoclonus clinically, its most common form, that is, hemifacial spasms, is easily recognized using needle EMG.

### Mimics of Myoclonus from Peripheral Disorders

The main peripheral myoclonus mimic is myokymia, in which a “true” peripheral origin can be identified. Myokymia classically results from radiation plexopathy or demyelination within either the spinal cord or the peripheral nervous system^106^, ^107^ and manifests as continuous, irregular quivering of muscles. With needle EMG, myokymic discharges are characterized by motor unit action potentials occurring as doublets, triplets, and multiplets, at inter‐burst frequencies of 2 to 10 Hz and intra‐burst spike frequency commonly between 5 and 62 Hz.^108^, ^109^ Therefore, the diagnosis of myokymia can be easily confirmed using needle EMG.

### Minipolymyoclonus

The term “minipolymyoclonus” or “polyminimyoclonus” refers to a hyperkinetic movement disorder characterized by intermittent, low‐amplitude, arrhythmic movements of the hands, commonly involving several fingers, with amplitudes just sufficient to produce visible and palpable movements of the joints.^110^ Minipolymyoclonus has been described in 3 main groups of neurological conditions: central neurodegenerative disorders, epilepsy, and diseases of spinal anterior horn cells. When associated with disorders causing cortical degeneration, minipolymyoclonus appears to have a similar pathophysiology to cortical myoclonus.^5^, ^9^, ^111^, ^112^, ^113^ For instance, several studies^114^, ^115^, ^116^ described patients affected by Parkinson's disease and multiple system atrophy presenting with minipolymyoclonus, which was characterized by variable combinations of brief EMG bursts (<100 ms), enhanced LLR, positive JLBA, and synchronous activation of agonist and antagonist muscles, with findings consistent with cortical myoclonus.^117^ Exceptions are represented by studies, such as in Alzheimer's disease, where minipolymyoclonus was preceded by a bifrontal EEG negativity, a finding interpreted as more suggestive of a subcortical, rather than cortical, generator.^118^

Minipolymyoclonus has been described in neurological disorders associated with epilepsy. Its electrophysiological features have been less well studied than in degenerative disorders, possibly because its cortical origin has been postulated to be due to the coexistence of epilepsy. This assumption has been partly confirmed,^28^, ^119^ as shown by electrophysiological features compatible with cortical myoclonus. An exception is the EEG discharges, which in some patients were bifrontal and long lasting (100–250 ms) and precedes the EMG burst associated with minipolymyoclonus by a highly variable interval (5–500 ms), sometimes too long to be considered cortical myoclonus.

Disease affecting the spinal motor neurons^110^, ^120^, ^121^, ^122^, ^123^, ^124^ or conditions causing peripheral nerve hyperexcitability, such as anti‐CASPR2‐associated paraneoplastic Morvan syndrome,^125^ have all been associated with minipolymyoclonus; however, accurate neurophysiological characterization in this context is lacking.

Inoue and colleagues^126^ described minipolymyoclonus, induced by voluntary movement, in a small number of patients affected by amyotrophic lateral sclerosis and found that EMG discharges associated with the jerks were compatible with large fasciculation potentials, which are caused by irregular activation of single motor units, either at the spinal motor neuron level or its axon.^108^ Another possibility, strengthened by the occurrence of minipolymyoclonus during movement, is that it may reflect unfused tetanus (sometimes named “contraction pseudotremor of chronic denervation”^127^). This can be observed when, as a result of chronic denervation, a reduced number of large motor units discharge at an abnormally high frequency.^110^, ^127^ Albeit not formally tested, the two entities should be easily discriminated by needle EMG: whereas fasciculations would show an irregular firing pattern and occur either spontaneously or during muscle activation, unfused tetanus would occur only during contraction and be associated with regular motor unit firing.

In conclusion, the term “minipolymyoclonus” does not indicate a single pathophysiological entity, and proposals have been made to use it only when its central origin is suspected, while resorting to “minipolyfasciculation” in the case of a peripheral origin.^123^, ^128^ Whereas the former would be sufficiently precise, the prefix “mini” in the latter term would likely be misleading, as the fasciculations causing minipolymyoclonus would necessarily be an expression of large motor unit discharges; therefore, “polyfasciculation” would likely be more appropriate. This phenomenon would still need to be distinguished from unfused tetanus, which may coexist but would represent a different entity.

### Jerky Functional Movement Disorders

Various neurophysiological features have been investigated in the context of jerky functional movement disorders^10^, ^129^; these include the inconsistency of the EMG pattern in relation to other types of myoclonus, the BP, event‐related desynchronization (ERD), and the auditory startle reflex.

Seven case series, involving 170 patients diagnosed with functional jerks, described the polymyographic pattern observed. The majority of patients exhibited an axial body distribution, specifically involving propriospinal, idiopathic spinal, and startle‐like reflex (n = 121), and the remainder palatal tremor/myoclonus (n = 9) or an unspecified presentation (n = 40).^22^, ^70^, ^71^, ^130^, ^131^, ^132^, ^133^, ^134^ The following findings were considered as “incongruent EMG”: (1) inconsistent activation of the first muscle, (2) inconsistent spread of muscle recruitment (rostral and caudal propagation), (3) burst duration >1000 ms, (4) no synchronous activation of agonist and antagonist, and (5) presence of distractibility or entrainment; and these were observed in approximately 75% of patients with clinical functional jerks.

The BP is the earliest movement‐related cortical potentials, namely EEG potentials that manifest around the time of movement, offering insights into the cortical activity underlying actions. BP is observed prior to a self‐paced movement and considered a signature of motor preparation^134^, ^135^; it is a slowly rising, negative cortical deflection started at least 1000 ms (early BP) or between 1000 and 500 ms (late BP) before the movement onset as measured using EMG.^10^ It has been shown that BP is present in 25% to 86% of patients with functional jerky movements (n = 216) and has a specificity of 100% in differentiating functional from “organic” myoclonus and a specificity of 86% differentiating functional myoclonus from tics.^22^, ^70^, ^71^, ^130^, ^131^, ^133^, ^136^, ^137^, ^138^, ^139^

ERD refers to reduction in amplitude of β and low γ EEG oscillations (13–45 Hz) prior to cued and self‐paced movements. Studies showed that ERD was present in 62% to 65% of patients with functional myoclonus (n = 49) and has a specificity of 100% in differentiating functional from cortical myoclonus.^137^, ^138^ The combination of BP and ERD has a sensitivity of 75% to 80% and a specificity of 100% in differentiating functional (n = 29) from cortical myoclonus.^137^

The auditory startle response has been studied in the context of functional jerks.^4^ A study showed increased response probability of the early and late phases of the startle reflex in patients with functional myoclonus (n = 17) compared to healthy controls.^131^ Furthermore, variable muscle recruitment was observed in the late phase.^131^

In summary, although electrophysiological techniques seem to be highly accurate in discriminating myoclonus from functional jerks, the existing evidence primarily derived from case series and a limited number of case–control studies. Moreover, technical challenges, such as the variability of the muscles involved in the jerky movements, should be considered as these features may limit the possibility of performing these tests based on signal averaging.

### Tics

Tics are brief and discrete movements that strongly resemble voluntary actions but occur repetitively and irregularly, and are not embedded in a discernible context.^140^

The variability in tic behaviors and their parallels to volitional motor behavior is also reflected in their physiological parameters as captured using surface EMG.^141^ Both EMG burst duration and pattern of muscle activation during tic do not provide unique clues to distinguish them from voluntary actions.^141^ Therefore, although surface EMG may be used to characterize tics, its application is not particularly useful for the purpose of diagnosis. Routine EEG recordings also do not provide reliable insights into the neural processes that underly tic generation.^142^ However, the combination of surface EMG and EEG with back‐averaging has been suggested to be useful in the distinction of tics from myoclonus, and in functional jerky movements that may resemble tics.^143^, ^144^ Several studies have used JLBA to demonstrate that some, but not all tics, may be preceded by BP.^136^, ^145^, ^146^, ^147^ Whereas the first study showed that tics were not preceded by BP,^146^ later studies reported that tics can indeed be preceded by BP, albeit with a short duration falling within the range of late BP, or normal BP.^136^, ^143^, ^145^, ^147^ Crucially, the morphology of the premovement potentials that precede tics often differs from phenomenologically similar voluntary actions.^136^, ^145^, ^146^, ^147^ Another neurophysiological cortical marker that has been examined is event‐related power changes, specifically ERD in the β frequency band. Several studies have reported an absence of β ERD with some tics,^148^, ^149^, ^150^ which is typically observed in volitional actions.^145^ A study examined both the presence of premotor potentials and event‐related β desynchronization preceding tics and reported a dissociation between the 2 phenomena in some cases.^145^

In conclusion, except for some clinico‐electrophysiological features (including the distribution and pattern of activation, absence of very short burst duration and negative bursts), the use of EMG is limited for the diagnosis of tics. Conversely, combined EEG–EMG recording, with back‐averaging technique (like BP) and event‐related power changes, might be helpful to distinguish tics from jerky functional movement disorders.

### Chorea

Although chorea is primarily characterized by a constant flow of movements that are randomly distributed across the body and over time, it is often described among jerky movement disorders.^151^ This is due to the nature of individual movements, which may still have a jerky quality. Distinguishing chorea from myoclonus can sometimes be challenging. EMG recordings of chorea typically show subcontinuous, fluctuating muscle activities of variable duration but often longer than that seen in myoclonus.^152^, ^153^ However, the diagnosis relies mainly on clinical presentation rather than neurophysiological studies, as there are a few studies on the CN of chorea.^10^

## Conclusion

Myoclonus is characterized by brief, jerky, shock‐like involuntary movements, which can result from sudden muscle contractions, known as positive myoclonus, or from the abrupt cessation of ongoing muscle activity, termed “negative myoclonus.”^154^ This straightforward definition can be readily translated into electrophysiological terms, as EMG recordings of myoclonus typically reveal brief bursts or interruptions in muscle activities. These features serve to differentiate myoclonus from other movement disorders, as EMG discharges in the myoclonus are too arrhythmic to be considered a tremor, too fast and brief to be dystonic or choreic, and not stereotyped as tics. Although these hallmarks are widely recognized, they have never been formally established, and the exceptions (eg, rhythmic myoclonus) are rarely considered, making the diagnosis or characterization of myoclonus using electrophysiology difficult at times. Another level of complexity is related to a peculiar aspect of myoclonus: it can originate from different parts of the central and peripheral nervous system, leading to variable clinical presentations. Myoclonus can indeed be focal, multifocal, or generalized, based on body distribution, and more or less brief based on the duration of EMG bursts; it is mostly arrhythmic, but it can be semirhythmic or rhythmic, and spontaneous, reflex, or action‐induced, depending on the provoking factors.^5^, ^61^, ^155^ Although these features depend on the source of its generation, a definitive clinico‐pathophysiological correlation remains elusive.

Our review showed that efforts to define myoclonus and jerky movement disorders using CN techniques have been made, but limitations persist due to a lack of systematic assessments and standardized methodologies. Only a small number of patients with jerky movement disorders assessed using CN techniques have been reported in the literature,^8^, ^34^ and conventional criteria for diagnosing myoclonus lack uniformity. For instance, whereas some techniques such as SEP are applied according to international guidelines,^156^ others, including JLBA, C‐reflex, and startle reflex, lack established methodologies, thereby hindering the comparison of results across different laboratories.^34^ A summary of the main clinical neurophysiological findings for each myoclonus subtype is presented in Table 4.

**TABLE 4:** Summary of the main clinical neurophysiological findings for each myoclonus subtype

	Cortical myoclonus	Myoclonus associated with dystonia	Brainstem (reticular) myoclonus	Excessive startle	Propriospinal myoclonus	Segmental myoclonus	Peripheral myoclonus	Jerky functional movement disorder
**Pattern of muscle activation**	Multifocal, arrhythmic (rarely rhythmic), stimulus sensitive	Jerks involving 1 muscle and its synergists, or co‐contraction of antagonist muscles; can be rhythmic	Early activation of trapezius and SCM followed by a rostral and caudal progression	Like brainstem myoclonus, but with auditory blink reflex early response; elicited by sensory stimuli (mainly auditory)	Flexor arrhythmic jerks of the axial and proximal limb muscles	Rhythmic or semirhythmic jerks (usually 1–3 Hz) in muscles innervated by the affected spinal segment	Rhythmic or pseudorhythmic jerks (1–3 Hz)	Inconsistent activation/spread of muscle recruitment, no synchronous activation of antagonist muscles, distractibility, entrainment
**EMG burst duration**	Usually short (<50 ms)	50–100 ms (up to 500 ms, which might reflect dystonic spasms)	10–100 ms (1 case up to 150 ms)	Longer EMG burst duration compared to brainstem myoclonus	50–300 ms (rarely 1000 ms or longer)	100–500 ms	Long (100–400 ms)	Very long burst duration (>1000 ms)
**SEP and C‐reflex**	Giant SEP and C‐reflex present	Generally, no giant SEP and no C‐reflex	C‐reflex in 2 of 8 cases	Enlarged/giant SEP in 2 patients	No giant SEP	No giant SEP		
**EEG–EMG**	JLBA and EEG–EMG coherence present	No JLBA and no EMG–EMG/EEG coherence			No EEG discharge BP present in many cases	No EEG discharge		BP present in 25–86% of the cases; specificity of 100%
**Others**	Duration up to 200 ms has been reported		Conduction velocity through the spinal cord is rapid	Conduction velocity through the spinal cord is moderately slow	Discharge slowly spread (5–15 m/s) rostrally and caudally the origin in spinal cord		In HFS, motor units fire in bursts (up to 40) at 200–300 Hz. There may be synchronicity between different muscles	ERD present in 62–65% of the cases; specificity of 100%
	Comments							
	Uncertainty persists regarding the inconsistent presence of the above markers	Small number of patients studied, but no clear signs of cortical involvement found	Small number of patients studied, but consistent pattern of muscle activation	Reproducible response and consistent pattern of muscle activation	Controversial. It is maybe rare, and most of the cases are likely caused by FMD	Extremely rare, only a few evidence available	Rare, it can be like segmental myoclonus. HFS is easily recognized using needle EMG	BP and ERD are highly accurate, but evidence is limited

Abbreviations: SCM, sternocleidomastoid; EMG, electromyography; SEP, somatosensory evoked potentials; EEG, electroencephalography; JLBA, jerk‐locked back averaging; BP, Bereitschaftspotential; HFS, hemifacial spasm; ERD, event‐related desynchronization; FMD, functional movement disorder.

The aforementioned limitations strongly warrant further studies to establish the sensitivity and specificity of each test, taking into consideration that the combination of these tests may increase the sensitivity.^34^ Moreover, standardization of both advanced and basic technical aspects, such as the number of epochs to record and average, EEG montage, EMG threshold for back‐averaging, automated process, and criteria for EMG burst, should be implemented.

In conclusion, CN may be regarded as the “gold standard” for defining myoclonus and at least some of its subtypes, significantly contributing to the diagnosis of jerky movement disorders. However, to enhance the quality and reliability of these tests, further research is warranted. This should encompass various myoclonus subtypes and other jerky movement disorders, involving larger cohorts of patients recruited from diverse centers. Standardized and optimized CN techniques should be employed to ensure consistency and validity across studies.

## Author Roles

1. Review: A. Conception, B. Organization, C. Execution
2. Manuscript preparation: A. Writing of the first draft, B. Review and critique

A.L.: 1A, B, C, 2A

C.G.: 1A, C, 2A

M.H.: 1A, C, 2A

N.P.: 1A, C, 2A

L.R.: 1A, C, 2A

S.M.: 2B

M.A.T.: 2B

S.V.: 1A, C, 2A

R.C.: 1A, B, C, 2B

## Disclosures

**Ethical Compliance Statement:** The authors confirm that the approval of an institutional review board was not required for this work. Informed patient consent was not necessary for this work. They confirm that they have read the journal's position on issues involved in ethical publication and affirm that this work is consistent with those guidelines.

**Funding Sources and Conflicts of Interest:** No specific funding was received for this work.

The authors declare that there are no conflicts of interest relevant to this work.

**Financial Disclosures for the Previous 12 Months:** A.L. is supported by EPSRC and MRC under the NEUROMOD+ Network (EP/W035057/1). She received honoraria from the Movement Disorder Society for educational activities. C.G. holds the Wolf Chair for Neurodevelopmental Psychiatry, a joint Hospital‐University Named Chair between the University of Toronto, the UHN, and the UHN Foundation. He received honoraria from the Movement Disorder Society for educational activities and academic research support from the VolkswagenStiftung (Freigeist Fellowship). M.A.T. reports grants from the Netherlands Organisation for Health Research and Development Domain: NWO‐TTW (2022‐9), ZonMW Topsubsidie (91218013), and ZonMW Program Translational Research (40‐44600‐98‐323). She also received 2 European Fund for Regional Development from the European Union (01492947) and DIMATIO (EFRO‐0059) and a European Joint Programme on Rare Diseases (EJP RD) Networking Support Scheme; a grant from the Health Holland and the PPP allowance program (PPP‐2023‐00). Furthermore, from the province of Friesland, she received the Stichting Wetenschapsfonds Dystonie and unrestricted grants from Ipsen, Actelion and Merz, STIL, AbbVie, and Teva. R.C. received honoraria from AbbVie, Merz, and Ipsen, and research grant from the Canadian Institutes of Health Research, the Natural Science and Engineering Research Council of Canada, Parkinson's Foundation, Dystonia Medical Research Foundation, and the National Organization for Rare Disorders.

## Data Availability

Data sharing is not applicable to this article as no new data were created or analyzed in this study.

## References

[1] Shibasaki H . Neurophysiological classification of myoclonus. Neurophysiol Clin 2006;36(5–6):267–269. 17336770 10.1016/j.neucli.2006.11.004

[2] Hallett M . Reappraisal of cortical myoclonus: electrophysiology is the gold standard. Mov Disord 2018;33(7):1190. 30153384 10.1002/mds.27430 PMC6116542

[3] Chen KS , Chen R . Principles of electrophysiological assessments for movement disorders. J Mov Disord 2020;13(1):27–38. 31986867 10.14802/jmd.19064 PMC6987526

[4] van der Veen S , Caviness JN , Dreissen YEM , et al. Myoclonus and other jerky movement disorders. Clin Neurophysiol Pract 2022;7:285–316. 36324989 10.1016/j.cnp.2022.09.003 PMC9619152

[5] Latorre A , Hale B , Rocchi L . How do I find clues about where myoclonus is originating? Mov Disord Clin Pract 2022;9(5):721–722. 35844279 10.1002/mdc3.13472 PMC9274365

[6] Pena AB , Caviness JN . Physiology‐based treatment of myoclonus. Neurotherapeutics 2020;17(4):1665–1680. 32910414 10.1007/s13311-020-00922-6 PMC7851206

[7] Caviness JN . Pathophysiology and treatment of myoclonus. Neurol Clin 2009;27(3):757–777. vii. 19555830 10.1016/j.ncl.2009.04.002

[8] Grippe T , Chen R . Utility of neurophysiological evaluation in movement disorders clinical practice. Mov Disord Clin Pract 2023;10:1599–1610. 10.1002/mdc3.13856 PMC10654828 38026509

[9] Latorre A , Rocchi L , Berardelli A , Rothwell JC , Bhatia KP , Cordivari C . Reappraisal of cortical myoclonus: a retrospective study of clinical neurophysiology. Mov Disord 2018;33(2):339–341. 29193325 10.1002/mds.27234

[10] van der Veen S , Klamer MR , Elting JWJ , Koelman J , van der Stouwe AMM , Tijssen MAJ . The diagnostic value of clinical neurophysiology in hyperkinetic movement disorders: a systematic review. Parkinsonism Relat Disord 2021;89:176–185. 34362669 10.1016/j.parkreldis.2021.07.033

[11] Zutt R , Elting JW , van Zijl JC , et al. Electrophysiologic testing aids diagnosis and subtyping of myoclonus. Neurology 2018;90(8):e647–e657. 29352095 10.1212/WNL.0000000000004996 PMC5818165

[12] Friedreich N. Paramyoclonus multiplex. Virchows Arch 1881;86:421–430.

[13] Grinker RR , Serota H , Stein SI . Myoclonic epilepsy. Arch Neurol Psychiatry 1938;40(5):968–980.

[14] Dawson GD . The relation between the electroencephalogram and muscle action potentials in certain convulsive states. J Neurol Neurosurg Psychiatry 1946;9(1):5–22. 20993388 10.1136/jnnp.9.1.5 PMC497916

[15] Halliday AM . Some observations from the study of evoked potentials in myoclonus epilepsy. Bratisl Lek Listy 1965;45(6):357–366. 5842772

[16] Shibasaki H , Kuroiwa Y . Electroencephalographic correlates of myoclonus. Electroencephalogr Clin Neurophysiol 1975;39(5):455–463. 52438 10.1016/0013-4694(75)90046-2

[17] Hallett M , Chadwick D , Marsden CD . Cortical reflex myoclonus. Neurology 1979;29(8):1107–1125. 572498 10.1212/wnl.29.8.1107

[18] Obeso JA , Rothwell JC , Marsden CD . The spectrum of cortical myoclonus. From focal reflex jerks to spontaneous motor epilepsy. Brain 1985;108(Pt 1): 193–224. 10.1093/brain/108.1.193 3919883

[19] Shibasaki H . AAEE minimonograph #30: electrophysiologic studies of myoclonus. Muscle Nerve 1988;11(9):899–907. 3050512 10.1002/mus.880110902

[20] Brown P , Farmer SF , Halliday DM , Marsden J , Rosenberg JR . Coherent cortical and muscle discharge in cortical myoclonus. Brain 1999;122(Pt 3):461–472. 10094255 10.1093/brain/122.3.461

[21] Grosse P , Guerrini R , Parmeggiani L , Bonanni P , Pogosyan A , Brown P . Abnormal corticomuscular and intermuscular coupling in high‐frequency rhythmic myoclonus. Brain 2003;126(Pt 2):326–342. 12538401 10.1093/brain/awg043

[22] Zutt R , Elting JW , van der Hoeven JH , Lange F , Tijssen MAJ . Myoclonus subtypes in tertiary referral center. Cortical myoclonus and functional jerks are common. Clin Neurophysiol 2017;128(1):253–259. 27940047 10.1016/j.clinph.2016.10.093

[23] Gasca‐Salas C , Arcocha J , Artieda J , Pastor P . Orthostatic myoclonus: an underrecognized cause of unsteadiness? Parkinsonism Relat Disord 2013;19(11):1013–1017. 23916653 10.1016/j.parkreldis.2013.07.004

[24] Terada K , Ikeda A , Mima T , et al. Familial cortical myoclonic tremor as a unique form of cortical reflex myoclonus. Mov Disord 1997;12(3):370–377. 9159732 10.1002/mds.870120316

[25] Brown P , Marsden CD . Rhythmic cortical and muscle discharge in cortical myoclonus. Brain 1996;119(Pt 4):1307–1316. 8813293 10.1093/brain/119.4.1307

[26] Brunt ER , Van Weerden TW , Pruim J , Lakke JW . Unique myoclonic pattern in corticobasal degeneration. Mov Disord 1995;10(2):132–142. 7753055 10.1002/mds.870100203

[27] Chen R , Ashby P , Lang AE . Stimulus‐sensitive myoclonus in akinetic‐rigid syndromes. Brain 1992;115(Pt 6):1875–1888. 1486465 10.1093/brain/115.6.1875

[28] Ikeda A , Kakigi R , Funai N , Neshige R , Kuroda Y , Shibasaki H . Cortical tremor: a variant of cortical reflex myoclonus. Neurology 1990;40(10):1561–1565. 2215948 10.1212/wnl.40.10.1561

[29] Shibasaki H , Yamashita Y , Neshige R , Tobimatsu S , Fukui R . Pathogenesis of giant somatosensory evoked potentials in progressive myoclonic epilepsy. Brain 1985;108(Pt 1):225–240. 3919884 10.1093/brain/108.1.225

[30] Ugawa Y , Shimpo T , Mannen T . Physiological analysis of asterixis: silent period locked averaging. J Neurol Neurosurg Psychiatry 1989;52(1):89–93. 2709041 10.1136/jnnp.52.1.89 PMC1032663

[31] Shibasaki H , Ikeda A , Nagamine T , et al. Cortical reflex negative myoclonus. Brain 1994;117(Pt 3):477–486. 8032858 10.1093/brain/117.3.477

[32] Obeso JA , Rothwell JC , Marsden CD . Somatosensory evoked potentials in myoclonus. Adv Neurol 1986;43:373–384. 3511630

[33] Ng K , Jones S . The "enhanced N35" somatosensory evoked potential: its associations and potential utility in the clinical evaluation of dystonia and myoclonus. J Neurol 2007;254(1):46–52. 17277914 10.1007/s00415-006-0237-5

[34] Latorre A , Belvisi D , Rothwell JC , Bhatia KP , Rocchi L . Rethinking the neurophysiological concept of cortical myoclonus. Clin Neurophysiol 2023;156:125–139. 37948946 10.1016/j.clinph.2023.10.007

[35] Bhatia KP , Bain P , Bajaj N , et al. Consensus statement on the classification of tremors. From the task force on tremor of the International Parkinson and Movement Disorder Society. Mov Disord 2018;33(1):75–87. 29193359 10.1002/mds.27121 PMC6530552

[36] Shibasaki H , Hallett M . Electrophysiological studies of myoclonus. Muscle Nerve 2005;31(2):157–174. 15547927 10.1002/mus.20234

[37] Obeso JA , Rothwell JC , Lang AE , Marsden CD . Myoclonic dystonia. Neurology 1983;33(7):825–830. 6683367 10.1212/wnl.33.7.825

[38] Kojovic M , Cordivari C , Bhatia K . Myoclonic disorders: a practical approach for diagnosis and treatment. Ther Adv Neurol Disord 2011;4(1):47–62. 21339907 10.1177/1756285610395653 PMC3036960

[39] Pollini L , van der Veen S , Elting JWJ , Tijssen MAJ . Negative myoclonus: neurophysiological study and clinical impact in progressive myoclonus ataxia. Mov Disord 2024;39(4):674–683. 38385661 10.1002/mds.29741

[40] Li JY , Cunic DI , Paradiso G , et al. Electrophysiological features of myoclonus‐dystonia. Mov Disord 2008;23(14):2055–2061. 18759341 10.1002/mds.22273

[41] Balint B , Guerreiro R , Carmona S , et al. KCNN2 mutation in autosomal‐dominant tremulous myoclonus‐dystonia. Eur J Neurol 2020;27(8):1471–1477. 32212350 10.1111/ene.14228

[42] Roze E , Apartis E , Clot F , et al. Myoclonus‐dystonia: clinical and electrophysiologic pattern related to SGCE mutations. Neurology 2008;70(13):1010–1016. 18362280 10.1212/01.wnl.0000297516.98574.c0

[43] Lavenstein B , McGurrin P , Attaripour S , Vial F , Hallett M . KCNN2 mutation in pediatric tremor myoclonus dystonia syndrome with electrophysiological evaluation. Tremor Other Hyperkinet Mov (N Y) 2022;12:2. 35106185 10.5334/tohm.668 PMC8796689

[44] Stamelou M , Mencacci NE , Cordivari C , et al. Myoclonus‐dystonia syndrome due to tyrosine hydroxylase deficiency. Neurology 2012;79(5):435–441. 22815559 10.1212/WNL.0b013e318261714a PMC3405253

[45] Foncke EM , Bour LJ , van der Meer JN , Koelman JH , Tijssen MA . Abnormal low frequency drive in myoclonus‐dystonia patients correlates with presence of dystonia. Mov Disord 2007;22(9):1299–1307. 17486590 10.1002/mds.21519

[46] Hallett M , Chadwick D , Adam J , Marsden CD . Reticular reflex myoclonus: a physiological type of human post‐hypoxic myoclonus. J Neurol Neurosurg Psychiatry 1977;40(3):253–264. 301926 10.1136/jnnp.40.3.253 PMC492659

[47] Brown P , Thompson PD , Rothwell JC , Day BL , Marsden CD . A case of postanoxic encephalopathy with cortical action and brainstem reticular reflex myoclonus. Mov Disord 1991;6(2):139–144. 1905387 10.1002/mds.870060209

[48] Rektor I , Kadanka Z , Bednarik J . Reflex reticular myoclonus: relationship to some brainstem pathophysiological mechanisms. Acta Neurol Scand 1991;83(4):221–225. 2048395 10.1111/j.1600-0404.1991.tb04686.x

[49] Clouston PD , Lim CL , Fung V , Yiannikas C , Morris JG . Brainstem myoclonus in a patient with non‐dopa‐responsive parkinsonism. Mov Disord 1996;11(4):404–410. 8813220 10.1002/mds.870110409

[50] Warren JD , Kimber TE , Thompson PD . Brainstem myoclonus in generalised tetanus. Mov Disord 2003;18(10):1204–1206. 14534931 10.1002/mds.10512

[51] Ong MT , Sarrigiannis PG , Baxter PS . Post‐anoxic reticular reflex myoclonus in a child and proposed classification of post‐anoxic myoclonus. Pediatr Neurol 2017;68:68–72. 28233665 10.1016/j.pediatrneurol.2016.12.014

[52] Beudel M , Elting JWJ , Uyttenboogaart M , van den Broek MWC , Tijssen MAJ . Reticular myoclonus: it really comes from the brainstem! Mov Disord Clin Pract 2014;1(3):258–260. 30713862 10.1002/mdc3.12054 PMC6353309

[53] Matsumoto J , Fuhr P , Nigro M , Hallett M . Physiological abnormalities in hereditary hyperekplexia. Ann Neurol 1992;32(1):41–50. 1642471 10.1002/ana.410320108

[54] Brown P , Rothwell JC , Thompson PD , Britton TC , Day BL , Marsden CD . The hyperekplexias and their relationship to the normal startle reflex. Brain 1991;114(Pt 4):1903–1928. 1884185 10.1093/brain/114.4.1903

[55] Tijssen MA , Padberg GW , van Dijk JG . The startle pattern in the minor form of hyperekplexia. Arch Neurol 1996;53(7):608–613. 8929168 10.1001/archneur.1996.00550070046011

[56] Tijssen MA , Voorkamp LM , Padberg GW , van Dijk JG . Startle responses in hereditary hyperekplexia. Arch Neurol 1997;54(4):388–393. 9109739 10.1001/archneur.1997.00550160034012

[57] Oguro K , Aiba H , Hojo H . Different responses to auditory and somaesthetic stimulation in patients with an excessive startle: a report of pediatric experience. Clin Neurophysiol 2001;112(7):1266–1272. 11516738 10.1016/s1388-2457(01)00568-5

[58] van de Warrenburg BP , Cordivari C , Brown P , Bhatia KP . Persisting hyperekplexia after idiopathic, self‐limiting brainstem encephalopathy. Mov Disord 2007;22(7):1017–1020. 17415799 10.1002/mds.21411

[59] Kiziltan ME , Gunduz A , Coskun T , et al. Startle response in progressive myoclonic epilepsy. Clin EEG Neurosci 2017;48(2):123–129. 27170668 10.1177/1550059416646292

[60] Brown P , Rothwell JC , Thompson PD , Britton TC , Day BL , Marsden CD . New observations on the normal auditory startle reflex in man. Brain 1991;114(Pt 4):1891–1902. 1884184 10.1093/brain/114.4.1891

[61] Merchant SHI , Vial‐Undurraga F , Leodori G , van Gerpen JA , Hallett M . Myoclonus: an electrophysiological diagnosis. Mov Disord Clin Pract 2020;7(5):489–499. 32626792 10.1002/mdc3.12986 PMC7328418

[62] Brown P , Thompson PD , Rothwell JC , Day BL , Marsden CD . Axial myoclonus of propriospinal origin. Brain 1991;114(Pt 1A):197–214. 1998882

[63] van der Salm SM , Erro R , Cordivari C , et al. Propriospinal myoclonus: clinical reappraisal and review of literature. Neurology 2014;83(20):1862–1870. 25305154 10.1212/WNL.0000000000000982 PMC4240434

[64] Kapoor R , Brown P , Thompson PD , Miller DH . Propriospinal myoclonus in multiple sclerosis. J Neurol Neurosurg Psychiatry 1992;55(11):1086–1088. 1469408 10.1136/jnnp.55.11.1086 PMC1015299

[65] Nogues M , Cammarota A , Sola C , Brown P . Propriospinal myoclonus in ischemic myelopathy secondary to a spinal dural arteriovenous fistula. Mov Disord 2000;15(2):355–358. 10752597 10.1002/1531-8257(200003)15:2<355::aid-mds1031>3.0.co;2-r

[66] Chung EJ , Kim SJ , Lee WY , Bae JS , Kim EG , Pang SH . Four cases with peripheral trauma induced involuntary movements. J Mov Disord 2010;3(2):39–41. 24868379 10.14802/jmd.10010 PMC4027669

[67] Facini C , Barsacchi M , Piccolo B , Turco EC , Pisani F . Early onset of propriospinal‐like myoclonus in a child following a vertebral fracture. Neurology 2016;87(9):956. 27572429 10.1212/WNL.0000000000003053

[68] Marrero‐Gonzalez P , Pascual‐Sedano B , Martinez‐Domeno A , et al. Isolated propriospinal myoclonus as a presentation of cervical myelopathy. Tremor Other Hyperkinet Mov (N Y) 2018;8:598. 30622837 10.7916/D8GQ8FNJ PMC6315061

[69] Sardana V , Sharma SK . Delayed propriospinal myoclonus following dorsal spinal cord surgery. Ann Indian Acad Neurol 2019;22(4):491–493. 31736579 10.4103/aian.AIAN_195_18 PMC6839301

[70] van der Salm SM , Koelman JH , Henneke S , van Rootselaar AF , Tijssen MA . Axial jerks: a clinical spectrum ranging from propriospinal to psychogenic myoclonus. J Neurol 2010;257(8):1349–1355. 20352254 10.1007/s00415-010-5531-6 PMC2910307

[71] Erro R , Bhatia KP , Edwards MJ , Farmer SF , Cordivari C . Clinical diagnosis of propriospinal myoclonus is unreliable: an electrophysiologic study. Mov Disord 2013;28(13):1868–1873. 24105950 10.1002/mds.25627

[72] Esposito M , Erro R , Edwards MJ , et al. The pathophysiology of symptomatic propriospinal myoclonus. Mov Disord 2014;29(9):1097–1099. 24976412 10.1002/mds.25951

[73] Brown P . Propriospinal myoclonus: where do we go from here? Mov Disord 2014;29(9):1092–1093. 24985526 10.1002/mds.25959

[74] Hoehn MM , Cherington M . Spinal myoclonus. Neurology 1977;27(10):942–946. 561907 10.1212/wnl.27.10.942

[75] Bagnato S , Rizzo V , Quartarone A , Majorana G , Vita G , Girlanda P . Segmental myoclonus in a patient affected by syringomyelia. Neurol Sci 2001;22(1):27–29. 11487188 10.1007/s100720170033

[76] Keswani SC , Kossoff EH , Krauss GL , Hagerty C . Amelioration of spinal myoclonus with levetiracetam. J Neurol Neurosurg Psychiatry 2002;73(4):457–458. 12235322 10.1136/jnnp.73.4.457 PMC1738068

[77] Warren JE , Vidailhet M , Kneebone CS , Quinn NP , Thompson PD . Myoclonus in spinal dysraphism. Mov Disord 2003;18(8):961–964. 12889092 10.1002/mds.10469

[78] Calancie B . Spinal myoclonus after spinal cord injury. J Spinal Cord Med 2006;29(4):413–424. 17044393 10.1080/10790268.2006.11753891 PMC1864857

[79] Davis SM , Murray NM , Diengdoh JV , Galea‐Debono A , Kocen RS . Stimulus‐sensitive spinal myoclonus. J Neurol Neurosurg Psychiatry 1981;44(10):884–888. 7310406 10.1136/jnnp.44.10.884 PMC491172

[80] Nanaura H , Kataoka H , Kiriyama T , et al. Spinal segmental myoclonus in both legs associated with antibodies to glycine receptors. Neurol Clin Pract 2019;9(2):176–177. 31041137 10.1212/CPJ.0000000000000557 PMC6461428

[81] Salman MS , Xu Q , Bunge M , Ilse W , Gerhold K , Udow SJ . Segmental myoclonus and epilepsy in a child with GAD 65 antibodies. Can J Neurol Sci 2022;49(1):136–139. 33557986 10.1017/cjn.2021.21

[82] Khatib L , Renard D , Honnorat J , Castelnovo G . Abdominal segmental myoclonus mimicking belly dancer dyskinesias in CASPR2 antibody encephalomyelitis. Mov Disord Clin Pract 2021;8(8):1260–1262. 36988995 10.1002/mdc3.13351 PMC8564816

[83] Tucker A , Kaul A , McKee J , et al. Surgical treatment of upper extremity segmental myoclonus in an adolescent with Chiari malformation and cervicothoracic syrinx. Pediatr Neurosurg 2021;56(4):373–378. 33975328 10.1159/000515519

[84] Kobayashi J , Yokochi F , Takasu M , Tobisawa S , Shimizu T . Spinal segmental myoclonus during postural maintenance in a patient with cervical spondylosis: a case report. Intern Med 2011;50(17):1839–1841. 21881285 10.2169/internalmedicine.50.5193

[85] Christodoulides I , Giamouriadis A , Bashford J , Barkas K . Spinal myoclonus: a rare presentation of cervical myelopathy. BMJ Case Rep 2018;2018:bcr2018225455. 10.1136/bcr-2018-225455 PMC6069946 30061134

[86] Pristas N , Klamar K , Napolitano J , Rosenberg N . Acute on chronic cervical myelopathy causing cervical segmental myoclonus in a high‐level wheelchair athlete: a case report. Spinal Cord Ser Cases 2021;7(1):90. 10.1038/s41394-021-00453-y PMC8481506 34588415

[87] Boudier‐Reveret M , Chang MC . Segmental spinal myoclonus after a cervical transforaminal epidural steroid injection. Am J Phys Med Rehabil 2020;99(11):e128–e130. 32149815 10.1097/PHM.0000000000001414

[88] Alroughani RA , Ahmed SF , Khan RA , Al‐Hashel JY . Spinal segmental myoclonus as an unusual presentation of multiple sclerosis. BMC Neurol 2015;15:15. 25879483 10.1186/s12883-015-0271-y PMC4347575

[89] McLaughlin AM , Khan TR , Lauritsen J , Batley K , Waugh JL . Segmental myoclonus following hepatorenal transplant and tacrolimus immunosuppression. Pediatr Neurol 2021;114:40–41. 33190072 10.1016/j.pediatrneurol.2020.09.008

[90] Berger JR , Bender A , Resnick L , Perlmutter D . Spinal myoclonus associated with HTLV III/LAV infection. Arch Neurol 1986;43(11):1203–1204. 2877649 10.1001/archneur.1986.00520110089026

[91] Dhaliwal GS , McGreal DA . Spinal myoclonus in association with herpes zoster infection: two case reports. Can J Neurol Sci 1974;1(4):239–241. 4441989 10.1017/s0317167100019831

[92] Roobol TH , Kazzaz BA , Vecht CJ . Segmental rigidity and spinal myoclonus as a paraneoplastic syndrome. J Neurol Neurosurg Psychiatry 1987;50(5):628–631. 3035105 10.1136/jnnp.50.5.628 PMC1031977

[93] Bhatia K , Thompson PD , Marsden CD . "isolated" postinfectious myoclonus. J Neurol Neurosurg Psychiatry 1992;55(11):1089–1091. 1469409 10.1136/jnnp.55.11.1089 PMC1015300

[94] Allen NM , Moran MM , King MD . Not all twitching is epileptic! Hand myoclonus in a boy with spinal cord tumor. J Pediatr 2013;162(2):431–431 e431. 22979978 10.1016/j.jpeds.2012.07.062

[95] Massimi L , Battaglia D , Paternoster G , Martinelli D , Sturiale C , Di Rocco C . Segmental spinal myoclonus and metastatic cervical ganglioglioma: an unusual association. J Child Neurol 2009;24(3):365–369. 19258299 10.1177/0883073808323027

[96] Pande S , Ang K , Myat MW , Neo S , Subramaniam S . Spinal segmental myoclonus following spinal surgery. Br J Neurosurg 2023;37(3):393–395. 32530327 10.1080/02688697.2020.1777262

[97] Levy R , Plassche W , Riggs J , Shoulson I . Spinal myoclonus related to an arteriovenous malformation. Response to clonazepam therapy. Arch Neurol 1983;40(4):254–255. 6830478 10.1001/archneur.1983.04050040084019

[98] Casazza M , Bracchi M , Girotti F . Spinal myoclonus and clinical worsening after intravenous contrast medium in a patient with spinal arteriovenous malformation. AJNR Am J Neuroradiol 1985;6(6):965–966. 3934939 PMC8333909

[99] Ross Russell AL , Knight W , Oware A , Fuller GN . A unique case of peripheral myoclonus. J Neurol Neurosurg Psychiatry 2013;84(1):117–118. 23184154 10.1136/jnnp-2012-303795

[100] Tyvaert L , Krystkowiak P , Cassim F , et al. Myoclonus of peripheral origin: two case reports. Mov Disord 2009;24(2):274–277. 19086086 10.1002/mds.21998

[101] Assal F , Magistris MR , Vingerhoets FJ . Post‐traumatic stimulus suppressible myoclonus of peripheral origin. J Neurol Neurosurg Psychiatry 1998;64(5):673–675. 9598689 10.1136/jnnp.64.5.673 PMC2170074

[102] Gregoire SM , Laloux P , Hanson P , Ossemann M , de Coene B . Segmental spinal myoclonus and syringomyelia: a case report. Acta Neurol Belg 2006;106(1):37–40. 16776436

[103] Marin Collazo IV , Tobin WO . Facial myokymia and hemifacial spasm in multiple sclerosis: a descriptive study on clinical features and treatment outcomes. Neurologist 2018;23(1):1–6. 29266036 10.1097/NRL.0000000000000163

[104] Wilkins RH . Hemifacial spasm: a review. Surg Neurol 1991;36(4):251–277. 1948626 10.1016/0090-3019(91)90087-p

[105] Hjorth RJ , Willison RG . The electromyogram in facial myokymia and hemifacial spasm. J Neurol Sci 1973;20(2):117–126. 4356328 10.1016/0022-510x(73)90025-7

[106] Albers JW , Allen AA 2nd , Bastron JA , Daube JR . Limb myokymia. Muscle Nerve 1981;4(6):494–504. 7311989 10.1002/mus.880040606

[107] Mateer JE , Gutmann L , McComas CF . Myokymia in Guillain‐Barré syndrome. Neurology 1983;33(3):374–376. 6681885 10.1212/wnl.33.3.374

[108] Bashford J , Chan WK , Coutinho E , Norwood F , Mills K , Shaw CE . Demystifying the spontaneous phenomena of motor hyperexcitability. Clin Neurophysiol 2021;132(8):1830–1844. 34130251 10.1016/j.clinph.2021.03.053

[109] Gutmann L . AAEM minimonograph #37: facial and limb myokymia. Muscle Nerve 1991;14(11):1043–1049. 1745276 10.1002/mus.880141102

[110] Spiro AJJN . Minipolymyoclonus: A neglected sign in childhood spinal muscular atrophy. Neurology 1970;20:1124–1126.

[111] Latorre A , Rocchi L , Cordivari C , Berardelli A , Bhatia KP , Rothwell JC . Reply: "reappraisal of cortical myoclonus: electrophysiology is the gold standard". Mov Disord 2018;33(7):1191. 30153388 10.1002/mds.27440

[112] Latorre A , Rocchi L , Magrinelli F , et al. Unravelling the enigma of cortical tremor and other forms of cortical myoclonus. Brain 2020;143(9):2653–2663. 32417917 10.1093/brain/awaa129

[113] Rocchi L , Latorre A , Ibanez Pereda J , et al. A case of congenital hypoplasia of the left cerebellar hemisphere and ipsilateral cortical myoclonus. Mov Disord 2019;34(11):1745–1747. 31609490 10.1002/mds.27881

[114] Salazar G , Valls‐Sole J , Marti MJ , Chang H , Tolosa ES . Postural and action myoclonus in patients with parkinsonian type multiple system atrophy. Mov Disord 2000;15(1):77–83. 10634245 10.1002/1531-8257(200001)15:1<77::aid-mds1013>3.0.co;2-n

[115] Okuma Y , Fujishima K , Miwa H , Mori H , Mizuno Y . Myoclonic tremulous movements in multiple system atrophy are a form of cortical myoclonus. Mov Disord 2005;20(4):451–456. 15593313 10.1002/mds.20346

[116] Caviness JN , Adler CH , Beach TG , Wetjen KL , Caselli RJ . Small‐amplitude cortical myoclonus in Parkinson's disease: physiology and clinical observations. Mov Disord 2002;17(4):657–662. 12210853 10.1002/mds.10177

[117] Zutt R , Elting JW , Tijssen MAJ . Tremor and myoclonus. Handb Clin Neurol 2019;161:149–165. 31307597 10.1016/B978-0-444-64142-7.00046-1

[118] Hallett M , Wilkins DE . Myoclonus in Alzheimer's disease and minipolymyoclonus. Adv Neurol 1986;43:399–405. 3946116

[119] Wilkins DE , Hallett M , Erba G . Primary generalised epileptic myoclonus: a frequent manifestation of minipolymyoclonus of central origin. J Neurol Neurosurg Psychiatry 1985;48(6):506–516. 3925089 10.1136/jnnp.48.6.506 PMC1028366

[120] McDonald CM . Clinical approach to the diagnostic evaluation of hereditary and acquired neuromuscular diseases. Phys Med Rehabil Clin N Am 2012;23(3):495–563. 22938875 10.1016/j.pmr.2012.06.011 PMC3482409

[121] Nalini A , Gourie‐Devi M , Thennarasu K , Ramalingaiah AH . Monomelic amyotrophy: clinical profile and natural history of 279 cases seen over 35 years (1976‐2010). Amyotrophic Lateral Scleros Frontotemporal Degener 2014;15(5–6):457–465. 10.3109/21678421.2014.903976 24853410

[122] Al‐Ghawi E , Al‐Harbi T , Al‐Sarawi A , Binfalah M . Monomelic amyotrophy with proximal upper limb involvement: a case report. J Med Case Reports 2016;10:54. 10.1186/s13256-016-0843-5 PMC4794906 26983673

[123] Bhat S , Ma W , Kozochonok E , Chokroverty S . Fasciculations masquerading as minipolymyoclonus in bulbospinal muscular atrophy. Ann Indian Acad Neurol 2015;18(2):249–251. 26019432 10.4103/0972-2327.150624 PMC4445210

[124] Nogués MA , Leiguarda RC , Rivero AD , Salvat F , Manes F . Involuntary movements and abnormal spontaneous EMG activity in syringomyelia and syringobulbia. Neurology 1999;52(4):823–834. 10078734 10.1212/wnl.52.4.823

[125] Vale TC , Pedroso JL , Dutra LA , et al. Morvan syndrome as a paraneoplastic disorder of thymoma with anti‐CASPR2 antibodies. Lancet 2017;389(10076):1367–1368. 28379152 10.1016/S0140-6736(16)31459-3

[126] Inoue M , Yamamoto M , Tsuzaki K , Hamano T , Etoh H , Shibasaki H . Large fasciculation can clinically manifest as spinal myoclonus; electromyographic and dynamic echomyographic studies of four cases with motor neuron disease. Clin Neurophysiol Pract 2018;3:6–10. 30214999 10.1016/j.cnp.2017.10.004 PMC6133775

[127] Riggs JE , Gutmann L , Schochet SS Jr . Contraction pseudotremor of chronic denervation. Arch Neurol 1983;40(8):518–519. 6870613 10.1001/archneur.1983.04210070058015

[128] Ganguly J , Chai JR , Jog M . Minipolymyoclonus: a critical appraisal. J Mov Disord 2021;14(2):114–118. 34062647 10.14802/jmd.20166 PMC8175814

[129] Thomsen BLC , Teodoro T , Edwards MJ . Biomarkers in functional movement disorders: a systematic review. J Neurol Neurosurg Psychiatry 2020;91(12):1261–1269. 33087421 10.1136/jnnp-2020-323141

[130] Esposito M , Edwards MJ , Bhatia KP , Brown P , Cordivari C . Idiopathic spinal myoclonus: a clinical and neurophysiological assessment of a movement disorder of uncertain origin. Mov Disord 2009;24(16):2344–2349. 19908306 10.1002/mds.22812

[131] Dreissen YEM , Boeree T , Koelman J , Tijssen MAJ . Startle responses in functional jerky movement disorders are increased but have a normal pattern. Parkinsonism Relat Disord 2017;40:27–32. 28410805 10.1016/j.parkreldis.2017.04.001

[132] Stamelou M , Saifee TA , Edwards MJ , Bhatia KP . Psychogenic palatal tremor may be underrecognized: reappraisal of a large series of cases. Mov Disord 2012;27(9):1164–1168. 22434706 10.1002/mds.24948 PMC4235251

[133] Vial F , Akano E , Attaripour S , McGurrin P , Hallett M . Electrophysiological evidence for functional (psychogenic) essential palatal tremor. Tremor Other Hyperkinet Mov (N Y) 2020;10:10. 32775024 10.5334/tohm.70 PMC7394203

[134] Shibasaki H , Hallett M . What is the Bereitschaftspotential? Clin Neurophysiol 2006;117(11):2341–2356. 16876476 10.1016/j.clinph.2006.04.025

[135] Hallett M . Neurophysiologic studies of functional neurologic disorders. Handb Clin Neurol 2016;139:61–71. 27719876 10.1016/B978-0-12-801772-2.00006-0

[136] van der Salm SM , Tijssen MA , Koelman JH , van Rootselaar AF . The bereitschaftspotential in jerky movement disorders. J Neurol Neurosurg Psychiatry 2012;83(12):1162–1167. 22952323 10.1136/jnnp-2012-303081

[137] Beudel M , Zutt R , Meppelink AM , et al. Improving neurophysiological biomarkers for functional myoclonic movements. Parkinsonism Relat Disord 2018;51:3–8. 29653908 10.1016/j.parkreldis.2018.03.029 PMC6022215

[138] Meppelink AM , Little S , Oswal A , et al. Event related desynchronisation predicts functional propriospinal myoclonus. Parkinsonism Relat Disord 2016;31:116–118. 27477621 10.1016/j.parkreldis.2016.07.010 PMC5120690

[139] Terada K , Ikeda A , Van Ness PC , et al. Presence of Bereitschaftspotential preceding psychogenic myoclonus: clinical application of jerk‐locked back averaging. J Neurol Neurosurg Psychiatry 1995;58(6):745–747. 7608681 10.1136/jnnp.58.6.745 PMC1073560

[140] Kurvits L , Martino D , Ganos C . Clinical features that evoke the concept of disinhibition in Tourette syndrome. Front Psychiatry 2020;11:21. 32161555 10.3389/fpsyt.2020.00021 PMC7053490

[141] Hallett M . Neurophysiology of tics. Adv Neurol 2001;85:237–244. 11530431

[142] Neufeld MY , Chistik V , Vishne TH , Korczyn AD . The diagnostic aid of routine EEG findings in patients presenting with a presumed first‐ever unprovoked seizure. Epilepsy Res 2000;42(2–3):197–202. 11074192 10.1016/s0920-1211(00)00183-2

[143] Panyakaew P , Cho HJ , Hallett M . Clinical neurophysiological evaluation for simple motor tics. Clin Neurophysiol Pract 2016;1:33–37. 27777987 10.1016/j.cnp.2016.04.001 PMC5074550

[144] McGurrin P , Attaripour S , Vial F , Hallett M . Purposely induced tics: electrophysiology. Tremor Other Hyperkinet Mov (N Y) 2020;10. 10.7916/tohm.v0.744 PMC7070698 32195038

[145] Triggiani AI , Scheman K , Pirio Richardson S , et al. Physiological and introspective antecedents of tics and movements in adults with tic disorders. Clin Neurophysiol 2023;151:143–150. 10.1016/j.clinph.2023.03.362 PMC10330543 37142497

[146] Obeso JA , Rothwell JC , Marsden CD . Simple tics in Gilles de la Tourette's syndrome are not prefaced by a normal premovement EEG potential. J Neurol Neurosurg Psychiatry 1981;44(8):735–738. 6946193 10.1136/jnnp.44.8.735 PMC491097

[147] Karp BI , Porter S , Toro C , Hallett M . Simple motor tics may be preceded by a premotor potential. J Neurol Neurosurg Psychiatry 1996;61(1):103–106. 8676135 10.1136/jnnp.61.1.103 PMC486469

[148] Gunduz A , Ganos C . Motor awareness, volition, and the cortical neurophysiology of simple motor tics. Clin Neurophysiol 2023;151:130–131. 10.1016/j.clinph.2023.04.005 37210248

[149] Morera Maiquez B , Jackson GM , Jackson SR . Examining the neural antecedents of tics in Tourette syndrome using electroencephalography. J Neuropsychol 2022;16(1):1–20. 33949779 10.1111/jnp.12245

[150] Cagle JN , Okun MS , Opri E , et al. Differentiating tic electrophysiology from voluntary movement in the human thalamocortical circuit. J Neurol Neurosurg Psychiatry 2020;91(5):533–539. 32139653 10.1136/jnnp-2019-321973 PMC7296862

[151] Abdo WF , van de Warrenburg BP , Burn DJ , Quinn NP , Bloem BR . The clinical approach to movement disorders. Nat Rev Neurol 2010;6(1):29–37. 20057497 10.1038/nrneurol.2009.196

[152] Bonomo R , Latorre A , Balint B , et al. Voluntary inhibitory control of chorea: a case series. Mov Disord Clin Pract 2020;7(3):308–312. 32258230 10.1002/mdc3.12907 PMC7111572

[153] Estevez‐Fraga C , Magrinelli F , Latorre A , et al. A new family with GLRB‐related hyperekplexia showing chorea in homo‐ and heterozygous variant carriers. Parkinson Relat Disord 2020;79:97–99. 10.1016/j.parkreldis.2020.08.016 32911248

[154] Marsden CD , Hallett M , Fahn S . The nosology and pathophysiology of myoclonus. In: S. MCDaF , ed. Movement Disorders. London: Butterworths; 1982:196–248.

[155] Shibasaki H , Thompson PD . Milestones in myoclonus. Mov Disord 2011;26(6):1142–1148. 21626558 10.1002/mds.23673

[156] Cruccu G , Aminoff MJ , Curio G , et al. Recommendations for the clinical use of somatosensory‐evoked potentials. Clin Neurophysiol 2008;119(8):1705–1719. 18486546 10.1016/j.clinph.2008.03.016

